# Systemic inflammation, lifestyle behaviours and dementia: A 10-year follow-up investigation

**DOI:** 10.1016/j.bbih.2024.100776

**Published:** 2024-04-22

**Authors:** Leah Hillari, Philipp Frank, Dorina Cadar

**Affiliations:** a Behavioural Science and Health, University College London, London, UK; b Department of Epidemiology and Biostatistics, School of Public Health, Imperial College London, London, UK; c UCL Brain Sciences, University College London, London, UK; d Centre for Dementia Studies, Brighton and Sussex Medical School, Brighton, UK

**Keywords:** Cognitive ageing, Dementia, Lifestyle behaviours, Prevention, Modifiable risk factors, Prospective cohort study, Mediation

## Abstract

**Objectives:**

Lifestyle behaviours have been linked to dementia incidence, but their cumulative impact on dementia and the underlying mechanisms remain poorly understood. This study investigated the association of co-occurring lifestyle behaviours with dementia incidence and the mediating role of systemic inflammation in this association.

**Methods:**

The sample comprised 3131 participants (55.2% female) from the English Longitudinal Study of Ageing aged 52–92 years at baseline (2008/09). Self-reported baseline lifestyle behaviours (alcohol intake, fruit and vegetable consumption, smoking, physical activity, sleep duration, social engagement, and cognitive activity) were summed to derive an index of lifestyle behaviours, ranging from 0 to 7, with higher scores denoting a higher number of health-risk behaviours. Incident dementia cases (n = 130, 4.2%) were identified through doctor-diagnosed dementia, informant interviews, and health records between 2014/15 and 2018/19. Systemic inflammation was measured through fasting plasma concentrations of C-reactive protein in 2012/13.

**Results:**

Binary logistic regression models indicated that the odds of subsequent dementia increased by 1.19 for each additional health-risk behaviour (95% confidence intervals: 1.04, 1.37, *p* = 0.014) after adjusting for age, sex, ethnicity, wealth, education, marital status, body mass index, coronary heart disease, hypertension, stroke, and depression. However, this association was not mediated by C-reactive protein.

**Conclusions:**

Co-occurring health-risk behaviours were associated with higher dementia incidence up to 10 years later, underscoring the importance of modifying health-risk behaviours for the prevention of dementia. Systemic inflammation did not explain the association between behaviours and dementia.

## 1 Introduction

Dementia is a syndrome characterised by progressive deterioration of the brain, which results in loss of cognitive function beyond what might be expected from normal ageing and severe enough to limit daily functioning (Sacuiu, 2016). Around 57 million people are living with dementia worldwide, a number estimated to reach nearly 153 by 2050 (Nichols et al., 2022).

As a fast-growing epidemic, dementia is responsible for an increasing proportion of mortality and morbidity worldwide. In 2023, dementia was the second most prevalent cause of mortality in the UK (Office for National Statistics, 2023) and is ranked amongst the top 10 causes of death globally (World Health Organization, 2023). Amongst individuals aged 60 years and older, dementia is one of the major contributors to disability and dependency, resulting in substantial years of healthy life lost (World Health Organization, 2021). Additionally, it places substantial care and financial burdens on family members, societies, and economies (Aranda et al., 2021). The global economic burden of dementia was estimated at US $1313.4 billion, with nearly half of the expenditure attributable to informal care (Wimo et al., 2023).

Given these considerable impacts and the absence of a cure or disease-modifying treatment (Tang et al., 2023), the prevention of dementia has become a global public health priority (Winblad et al., 2016). Meta-analyses and systematic reviews of longitudinal studies have identified multiple behavioural predictors of dementia that could potentially be targeted for its prevention. High fruit and vegetable consumption (Jiang et al., 2017), low-to-moderate alcohol intake (Rehm et al., 2019), and sleeping 7–8 h per night (Wu et al., 2018) have been found to protect against dementia. Conversely, smoking (Zhao et al., 2021) and low participation in mental, physical, or social activity (Fratiglioni et al., 2020) have been shown to increase dementia risk.

While the individual impact of lifestyle behaviours has been widely investigated, few studies have examined their cumulative impact on dementia risk (Livingston et al., 2020; Zhang et al., 2022). Such studies are urgently needed given that health-risk behaviours, including physical inactivity, excess alcohol intake, poor diet, and smoking, do not exist in isolation but tend to co-occur and cluster among individuals (Meader et al., 2016). Assessments of individual health-risk behaviours might underestimate total dementia risk as they only index small proportions of health-risk behavioural clusters (Whalley et al., 2006). Equally plausible, these assessments might overestimate dementia risk as the impact of individual health-risk behaviours might be cancelled out by co-existing health-protective behaviours (Hessler et al., 2016). Thus, accurate prediction of modifiable lifestyle risk requires consideration of the multitude of brain-relevant behaviours performed in daily life.

To date, only a few studies have employed a multi-behaviour approach for dementia risk prediction. These studies consistently documented prospective associations between lower behavioural risk and lower subsequent incidence of all-cause dementia and Alzheimer's dementia (AD) (Dhana et al., 2020; Norton et al., 2012; Scarmeas et al., 2009). For instance, in two prospective cohort studies comprising 1880 community-dwelling adults living in the United States of America (USA) (mean age 77 years), adherence to a Mediterranean-type diet and regular physical activity was associated with a lower risk of AD after approximately five years, compared to non-adherence to the diet and physical inactivity (Scarmeas et al., 2009). The most recent study demonstrated that, for each additional protective behaviour (non-smoking, moderate/vigorous-intensity physical activity, low-to-moderate alcohol consumption, high-quality diet, cognitive activity), AD risk was reduced by 27% after about six years among 2765 individuals (mean age 77 years) enrolled in the Chicago Health and Ageing Project and the Rush Memory and Aging Project (Dhana et al., 2020).

While providing consistent evidence for the predictive role of co-occurring behaviours in AD and all-cause dementia, these studies were limited by several shortcomings. Firstly, they employed relatively short follow-up periods of up to six years, raising the question of whether observed associations might be due to reverse causality bias (i.e., lifestyle behaviours might result from preclinical dementia symptomatology rather than cause dementia development) (Weuve et al., 2015). Secondly, all studies were conducted in the US, limiting the generalisability of findings to other peer countries in which lifestyle risk and chronic disease prevalence are lower. Thirdly, all studies lacked the concurrent inclusion of cognitive activity, social engagement, and sleep duration, which have emerged as robust behavioural predictors of dementia over the past years (Livingston et al., 2020). Lastly, except for one study (Norton et al., 2012), all studies investigated AD risk, although all-cause dementia may have provided a more valid outcome as many dementia cases are of’ mixed ‘presentation with converging Vascular dementia (VAD)- and AD-related pathologies (De Reuck et al., 2016). Given these limitations, the cumulative impact of lifestyle behaviours on all-cause dementia incidence remains inconclusive and warrants further investigation.

Further research is also required to identify the exact biological pathways by which co-occurring lifestyle behaviours “get under the skin” to affect brain health. Thus far, these mechanisms remain inadequately elucidated (MacKinnon and Luecken, 2008). Identifying such pathways may help determine the biological plausibility of lifestyle behaviours in dementia development and their value as prevention targets (Olsen and Jensen, 2019). Systemic inflammation may represent a shared biological mechanism through which lifestyle behaviours affect dementia development. Systemic inflammation hereinafter referred to as inflammation, describes a non-resolving, elevated concentration of pro-inflammatory markers in the peripheral nervous system, such as the cytokines tumour necrosis factor α (TNF-α) and interleukin-1β (IL-1β), and the acute-phase protein c-reactive protein (CRP) (Pietzner et al., 2017).

Inflammation can affect brain health as it can spread from peripheral tissue to the central nervous system (CNS), where it can initiate a state of neuroinflammation (Cervellati et al., 2020). In contrast to acute brain inflammation, neuroinflammation denotes a chronic inflammatory state of the CNS characterised by sustained glial cell activation and immune cell infiltration into the CNS (Streit et al., 2004). Neuroinflammation is known to initiate and exacerbate β-amyloid oligomerisation, tau hyperphosphorylation, and neuronal and synaptic loss, which may eventually lead to neurodegenerative dementias such as AD (Pasqualetti et al., 2015). Due to their atherogenic and prothrombotic nature, peripheral inflammatory markers can also compromise endothelial and vascular function. Thereby, they promote the occurrence of brain infarcts, haemorrhages, and microvascular lesions, the most common causes of VAD (Wolters and Ikram, 2019). This is in line with the results of a recent meta-analysis of 13 studies showing a robust association between elevated levels of pro-inflammatory markers such as CRP and α1-antichymotrypsin and all-cause dementia (Darweesh et al., 2018).

The potential adverse effects of inflammation on brain health are particularly concerning as biological ageing is characterised by increasing levels of pro-inflammatory markers, a phenomenon termed “inflammageing” (Franceschi and Campisi, 2014). While driven by the ageing process, inflammageing might be reduced by adhering to a healthy lifestyle (Ruiz-Núñez et al., 2013). Indeed, cross-sectional studies linked socio-cognitive engagement (e.g., volunteering, attending meetings) (Kim and Ferraro, 2014; Smith et al., 2020) and low-to-moderate alcohol intake (Attard et al., 2021) to lower levels of peripheral, pro-inflammatory markers. Moreover, interventional trials have demonstrated the anti-inflammatory effects of physical activity (Sellami et al., 2021), healthy eating (Neale et al., 2016) and smoking cessation (Gallus et al., 2018), and pro-inflammatory effects of sleep restriction (Mullington et al., 2010). Based on this evidence, the present study suggests that co-occurring lifestyle behaviours might be linked to subsequent dementia incidence through their impact on inflammation.

## 2 Aims and hypotheses

To fill the research gap concerning the cumulative role of lifestyle behaviours in predicting all-cause dementia, the present study investigated the association between seven co-occurring lifestyle behaviours and subsequent dementia status. By examining the potential inflammatory pathway explaining this association, it addressed a further research gap.

It was hypothesised that:

1. A higher number of health-risk lifestyle behaviours would be associated with increased odds of developing dementia up to 10 years later (c, Fig. 1).

**Fig. 1:**
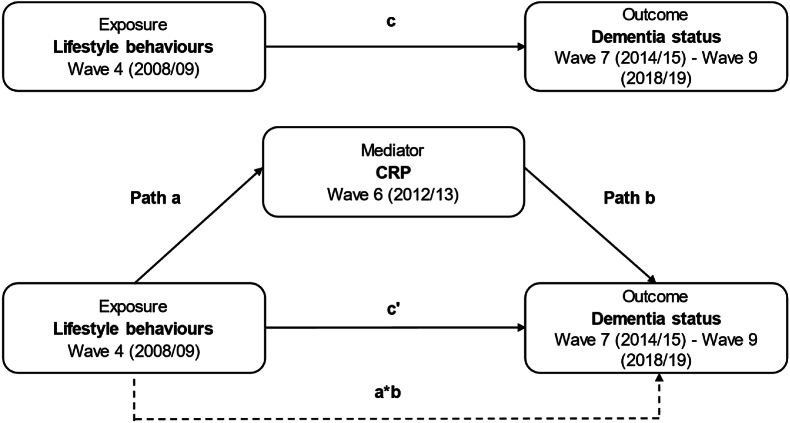
A conceptual figure of the mediating role of c-reactive protein (Wave 6) in the association of baseline lifestyle behaviours (Wave 4) and dementia status (Wave 7–9). *Note.* CRP = C-reactive protein Coefficient a = The coefficient for the ILB in a model predicting CRP from the ILB Coefficient b = The coefficient for CRP in a model predicting dementia status from CRP (adjusted for baseline ILB scores) c' = Direct effect = The effect of the ILB on dementia (adjusted for CRP as mediator) a*b = Indirect effect = The effect of the ILB on dementia via CRP c = Total effect = The effect of the ILB on dementia.
2. Inflammation (indexed by CRP) would mediate the association between baseline health-risk lifestyle behaviours and subsequent incident dementia (a*b, Fig. 1).

## 3 Methods

### 3.1 Design

Prospective cohort study.

### 3.2 Participants

Data were drawn from the English Longitudinal Study of Ageing (ELSA), an ongoing, population-based, prospective cohort study (Steptoe et al., 2013). ELSA participants were recruited from the Health Surveys for England, a selection of annual, population-based surveys carried out in 1998, 1999, and 2001. At inception in 2002–2003, the ELSA core sample comprised 11,391 nationally representative males and females aged ≥50 years who were living in private residential households in England (individual response rate 67%) (Marmot et al., 2003). Since the inception, the ELSA sample has been followed up biannually. At every wave, self-report data were collected face-to-face via computer-assisted personal interviewing (CAPI) and self-completion questionnaires. On alternate waves, biomedical measures were obtained during nurse visits at participants' homes.

**Fig. 2:**
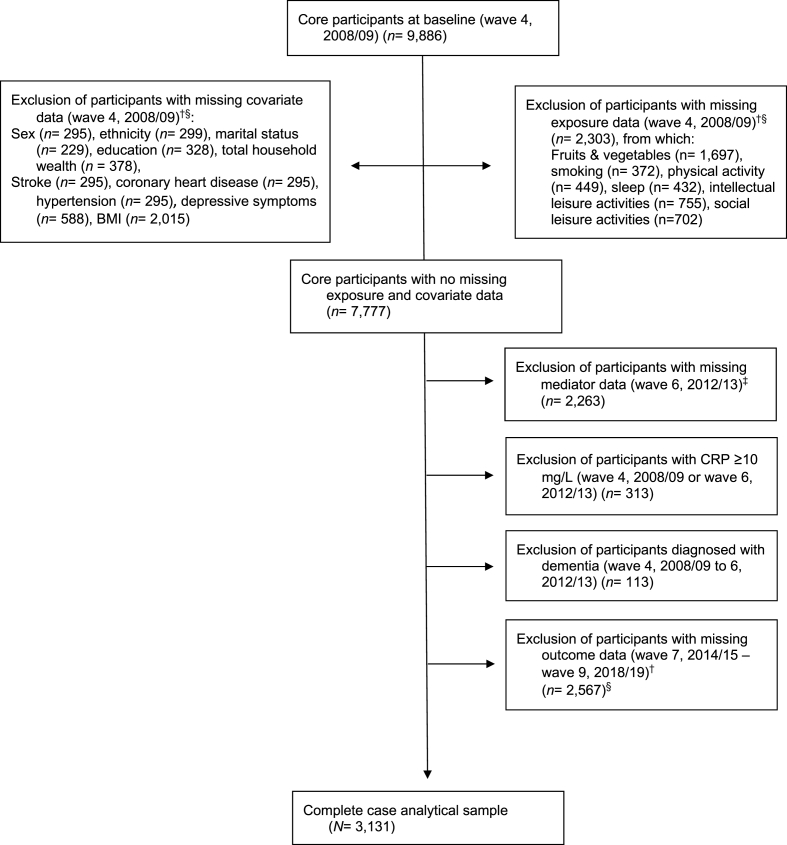
Participant flow-chart *Note.* Body mass index = BMI; CHD= Coronary Heart Disease; CRP= C-reactive protein ^†^ Reasons for missing data were ‘terminating events' such as moves out of Great Britain, institutional moves, death, or non-response (owing to refusal and inability to trace participants). ^‡^ Biological data was missing for participants with clotting or bleeding disorders, taking anticoagulant medication, not willing to give their written consent or having had fits or convulsions (NatCen Social Research, 2018). ^§^ Non-mutually exclusive.

Of the 9886 ELSA members participating in wave 4, those with dementia and those with invalid or missing exposure, covariate, mediator, or outcome data were omitted from the analytical sample. To ensure that the mediator (CRP measured at wave 6) preceded the dementia outcome, participants with a dementia diagnosis by wave 6 were excluded. Additionally, participants with baseline levels of CRP ≥10 mg/L at wave 4 and/or wave 6 were excluded as these levels indicate acute inflammation due to infection/injury rather than systemic, non-resolving inflammation (Markanday, 2015). The described selection process resulted in a complete case analytic sample of 3131 adults (see Fig. 2).

ELSA was carried out in accordance with the Declaration of Helsinki, and ethical approval was received from the London Multicentre Research and Ethics Committee (reference number: MREC/01/2/91). All participants provided informed consent prior to every ELSA wave. The strengthening of the reporting of observational studies in epidemiology guidelines was followed for this article (von Elm et al., 2014).

### 3.3 Materials and procedure

#### 3.3.1 Measures

**Exposure Variable – Index of Lifestyle Behaviours.** Seven lifestyle behaviours were selected due to their established relationships with dementia (Deckers et al., 2015; Livingston et al., 2020) and inflammation (Kim and Ferraro, 2014; Loprinzi, 2016). Each behaviour (presented below) was self-reported at baseline, wave 4 (2008/09), as this was when the sleep measure was introduced in ELSA. For each behaviour, participants were assigned a score of 0 for a health-protective lifestyle behaviour or 1 for a health-risk lifestyle behaviour. This classification was based on guidelines, recommendations, and prior evidence, as detailed in the supplementary material (Table S1). All behavioural variables were summed to derive an index of lifestyle behaviours (ILB), ranging from 0 to 7, with higher scores denoting a higher number of health-risk behaviours. The ILB was considered as a continuous variable as its categorisation might have resulted in a loss of precision and power (Bennette and Vickers, 2012).

***Alcohol Intake.*** Respondents were asked how often they had an alcoholic drink in the last 12 months. The validity of this self-report measure has been confirmed in previous population-based studies.

***Fruit and Vegetable Consumption.*** Participants were asked about the number of various-sized fruits, fruit juices, salads, tablespoons of vegetables (excluding potatoes), pulses, and dishes mainly made from fruits or vegetables consumed on the previous day. The quantity of consumed fruits and vegetables was transformed into portions (1 portion = 80g fruits/vegetables) according to definitions by the National Health Service (National Health Service, 2018) (see Fig. S1 for conversion rates). Finally, a summary variable representing the total portions of fruits and vegetables eaten in one day was computed.

***Smoking status.*** Participants were asked whether they had ever smoked (“yes/no”), and if they answered affirmatively, whether they still smoked.

***Physical Activity.*** Participants were asked how frequently they engaged in leisure-time sports/activities. Activities were classified in terms of intensity: mild, moderate, and vigorous, which corresponds to metabolic equivalent (MET) scores of ≥2 to <3.5, ≥3.5 to <6, and ≥6, respectively (Ainsworth et al., 2000). MET scores indicate the energy consumption of specific physical activities as multiples of the standard resting metabolic rate calibrated during quiet sitting.

***Sleep Duration.*** Sleep duration was ascertained through self-reported average sleep duration (to the nearest half-hour) on a weeknight.

***Social Engagement & Cognitive Activity.*** Social engagement and cognitive activity were assessed based on two aggregated scores reflecting the total number of self-reported occasions of participation in social and intellectual leisure activities, respectively (see Table S1 for included activities). The creation of these scores has been described previously (Almeida-Meza et al., 2021).

**Outcome Variables – Dementia Status.** Participants were classified as dementia cases if they met any of the following criteria. The first criterion was a doctor-diagnosis of dementia or AD reported by the participant or an informant (e.g., caregiver, family member) between waves 7 and 9. The second criterion was dementia or AD diagnosis established through participants' electronic health records obtained from the Hospital Episode Statistics (HES) database for England up to wave 8 (2016/17) (NHS Digital, 2021). In HES, dementia diagnoses are documented as per the International Statistical Classification of Diseases and Related Health Problems, 10th Revision codes (World Health Organization, 2004), with moderate sensitivity (78%) and high specificity (92%) (Sommerlad et al., 2018). The third criterion was an average score above 3.38 on the adapted 16-item Informant Questionnaire on Cognitive Decline in the Elderly (IQCODE) administered at wave 9 (Jorm, 1994). This IQCODE is a widely used, validated scale which is administered to an informant to assess participants' present functional and cognitive performance compared to their performance over the previous two years (Park, 2017). The threshold of 3.38 has high specificity (84%) and sensitivity (82%) for detecting all-cause dementia in community-dwelling older adults (Quinn et al., 2014).

**Mediator Variables – CRP.** The acute-phase protein CRP is one of the most extensively used biomarkers of inflammation. Fasting plasma concentrations of CRP (in mg/L) were assayed through 6 ml of blood taken from participants during nurse visits at wave 6 (2012/13). CRP concentrations were assayed at the Royal Victoria Infirmary laboratory in Newcastle (UK) with the N Latex CRP mono Immunoassay on the Behring Nephelometer II Analyser (Dade Behring, Milton Keynes, UK).

**Covariates.** Covariates were selected based on theory and evidence. Except for weight and height measurements taken during nurse visits, all covariates were self-reported. Covariates included age (in years), sex (“female”, “male”) and marital status (“married/remarried”, “divorced/legally separated”, “widowed”, “single”). As current participants were predominantly White Caucasian (98.5%), ethnicity was categorised into “White” and “other ethnic group”. Education level and wealth were assessed as robust indicators of socioeconomic position (Marmot et al., 2003). Total household wealth was determined by summing participants' financial, housing, and physical wealth, as well as net of debt. It was divided into quintiles, with the fifth quintile denoting the highest wealth category. Education level was classified into “low” (no formal qualification), “intermediate” (qualifications awarded at the end of state-regulated schooling), and “higher” (below degree or degree-level qualification).

The presence of stroke, coronary heart disease (CHD), and hypertension was determined based on participants' or informants' reporting of a doctor's diagnosis. Depressive symptoms were measured using the validated, 8-item version of the Centre for Epidemiological Studies Depression Scale (CES-D) (Radloff, 1977). A well-recognised threshold value of ≥3 indicated elevated levels of depressive symptoms.

Weight and height assessments were used to compute body mass index (BMI) (kg/m^2^), which was subsequently categorised in accordance with the WHO definitions of underweight (<18.5 kg/m^2^), normal weight (18.5–24.9 kg/m^2^), overweight (25.0–29.9 kg/m^2^), and obesity (≥30.0 kg/m^2^) (World Health Organization, 2000). The underweight and normal weight categories were collapsed due to infrequent cases in the underweight category.

### 3.4 Analyses

Baseline characteristics of complete case participants' overall and stratified by dementia status at follow-up are reported as frequencies and percentages for categorical variables and means and standard deviations for continuous variables. Differences in baseline characteristics between those who remained dementia-free and those who developed dementia during follow-up were analysed through independent-sample *t*-tests for continuous variables, Fisher's exact tests and Pearson Chi-square tests for categorical variables.

Binary logistic regression analyses were used to investigate the association of the ILB with dementia status at follow-up. After fitting an unadjusted model (*Model 1*), covariates were included in a stepwise fashion (Model 2: adjusted for age, sex, ethnicity, marital status; Model 3: Model 2 & further adjusted for total household wealth, education; Model 4: Model 3 & further adjusted for BMI, CHD, hypertension, stroke, depression).

Mediation analyses were used to test whether the association between the ILB and dementia status was mediated by CRP (see Fig. 1). The PROCESS package for SPSS (Hayes, 2018) was used to quantify the mediating effect of CRP (indirect effect) while controlling for all covariates. The Wave 4 and 6 CRP variables (Kurtosis >106.35, skewness >20.54) were log-transformed to normalise the distribution of residuals. Results of the binary logistic regression models are presented as odds ratios (OR) with corresponding 95% confidence intervals (CI). For the mediation analyses, indirect effects are presented with Bias-corrected (Bc) 95% CI based on 1000 bootstrap samples. All other mediation results are presented as unstandardised coefficients (B) and 95% CI. Bc 95% CI were used to infer the statistical significance of indirect effects. For all other results, statistical significance was defined using a two-sided *p*-value set at <0.05.

We re-estimated the logistic regression model with an LBI-sex interaction term and tested whether the main mediation model was moderated by sex (moderated mediation analysis, model 59, in PROCESS) to examine the presence of sex-specific effects and the necessity to perform sex-stratified analyses. All analyses were performed using IBM's SPSS, Version 27.0 (Armonk, NY: IBM Corp).

#### 3.4.1 Sensitivity analysis

The first sensitivity analysis re-estimated the initial mediation model, including baseline CRP levels as a covariate to test the temporal precedence of the exposure (lifestyle behaviours) relative to the mediator (inflammation), allowing causal inference about the assumed effect of the exposure on the mediator. The second sensitivity analysis re-estimated the main mediation model, including participants with CRP values ≥ 10 mg/L since high CRP levels can be observed even in non-resolving, systemic inflammation. The third sensitivity analysis stratified the initial mediation model by baseline age groups (50–65 years vs.>65 years) since lifestyle factors have shown age-dependent effects (Deckers et al., 2020). The final sensitivity analysis added diabetes as a covariate to the initial mediation model due to the established links between diabetes and lifestyle behaviour (Khan et al., 2023), inflammation (Wang et al., 2013) and dementia (Livingston et al., 2020). Diabetes was determined based on participants' or informants' reporting of a doctor's diagnosis. A detailed description of the sensitivity analyses can be found on p. 3 of the supplementary material.

## 4 Results

### 4.1 Baseline participants' characteristics

Table 1 displays the baseline characteristics of the complete case analytical sample, comprising 3131 participants, of whom 1727 (55.2%) were female and 1404 (44.8%) were male. Participants' age ranged from 50 to 92 years (*M* = 59.64 years, *SD* = 7.20). The ILB was approximately normally distributed (Kurtosis: −0.03, skewness: 0.67) with a mean of 1.86 (*SD* = 1.42). During the 5-10-year follow-up period, 130 (4.2%) cases of incident all-cause dementia were recorded.

**Table 1:** Baseline characteristics of the analytic sample overall (*N* = 3131) and stratified by dementia status.

Variables	All (*N* = 3131)	No dementia (*n* = 3001)	Dementia (*n* = 130)	*P*-value
***Baseline predictor***				
ILB, mean (SD)	1.86 (1.42)	1.82 (1.41)	2.50 (1.52)	0.001^a^
***Baseline covariates***				
Age (years), mean (SD)	59.64 (7.20)	59.64 (7.21)	66.72 (8.43)	0.001^c^
Sex, *n* (*%*)				0.553^b^
Female	1727 (55.2)	1652 (55.0)	75 (57.7)	
Male	1404 (44.8)	1349 (45.0)	55 (42.3)	
Ethnicity, *n* (%)				0.971^d^
White	3084 (98.5)	2956 (98.5)	128 (98.5)	
Other	47 (1.5)	45 (1.5)	2 (1.5)	
Marital status, n (%)				<0.001^b^
Married/remarried *(ref)*	2295 (73.3)	2216 (73.8)	79 (60.8)	
Divorced/legally separated	370 (11.8)	357 (11.9)	13 (10.0)	0.512^b^
Widowed	272 (8.7)	241 (8.0)	31 (23.8)	0.001^b^
Single	194 (6.2)	187 (6.2)	7 (5.4)	0.695^b^
Total household wealth, *n* (*%*)				<0.001^b^
1^st^ quintile (lowest) *(ref)*	294 (9.4)	269 (9.0)	25 (19.2)	
2^nd^ quintile	522 (16.7)	495 (16.5)	27 (20.8)	0.200^b^
3^rd^ quintile	648 (20.7)	622 (20.7)	26 (20.0)	0.841^b^
4^th^ quintile	724 (23.1)	695 (23.2)	29 (22.3)	0.822^b^
5^th^ quintile (highest)	943 (30.1)	920 (30.7)	23 (17.7)	0.002^b^
Education level, *n* (*%*)				<0.001^b^
Low *(ref)*	774 (24.7)	727 (24.2)	47 (36.2)	
Intermediate	1240 (39.6)	1183 (39.4)	57 (43.8)	0.312^b^
Higher	1117 (35.7)	1091 (36.4)	26 (20.0)	<0.001^b^
BMI (kg/m^2^), *n* (*%*)				0.390^b^
UW/NW (<24.9) (ref)	900 (28.7)	866 (28.9)	34 (26.2)	
Overweight (25.0–29.9)	1383 (44.2)	1318 (43.9)	65 (50.0)	0.396^b^
Obese (≥30.0)	848 (27.1)	817 (27.2)	31 (23.8)	0.172^b^
CES-D Depressive Symptoms, *n (%)*				<0.001^b^
Elevated (CES-D score ≥3)	528 (16.9)	492 (16.4)	94 (72.3)	
Normal (CES-D score <3)	2603 (83.1)	2509 (83.6)	36 (27.7)	
CHD, *n* (*%*)				0.019^b^
Yes	136 (4.3)	125 (4.2)	11 (8.5)	
No	2995 (95.7)	2876 (95.8)	119 (91.5)	
Hypertension, *n* (*%*)				0.003^b^
Yes	623 (19.9)	584 (19.5)	39 (30.0)	
No	2508 (80.1)	2417 (80.5)	91 (70.0)	
Stroke, *n* (*%*)				0.137^c^
Yes	3099 (99.0)	29 (1.0)	3 (2.3)	
No	32 (1.0)	2972 (99.0)	127 (97.7)	
CRP (mg/L)^d^, mean (*SD*)	2.29 (2.06)	2.29 (2.06)	2.28 (2.19)	0.949^a^

*Note. SD*= Standard deviation; ILB = Index of lifestyle behaviours; BMI= Body mass index; UW= Underweight; NW= Normal weight; CES-D = Centre for Epidemiologic Studies Depression Scale; CHD= Coronary heart disease; CRP=C-reactive protein.

Fisher's exact test (calculated if expected cell frequencies were smaller than five for Pearson Chi-square tests).

a Independent-samples *t*-test.

b Pearson's chi-square test.

c Greenhouse-geisser corrected *p*-value (calculated if the assumption of sphericity were violated for the independent-samples *t*-tests).

d Untransformed data.

At baseline, participants who developed dementia were older, scored higher on the ILB, were more likely to be in the lowest quintile of total household wealth, more likely to have elevated levels of depressive symptoms, CHD, hypertension, low education, but were less likely to be married, compared to those who remained dementia-free (all *p* < 0.05).

### 4.2 Association between lifestyle behaviours and dementia status

The parametric assumptions of logistic regression were met. Table 2 displays the results from the binary logistic regression models. The unadjusted model (*Model 1*) showed that a 1-unit increase in the ILB was related to 1.35 higher odds of developing dementia within the following 5–10 years (95% CI: 1.20, 1.50, *p* < 0.001). This association was attenuated to 1.29 higher odds but remained significant in *Model 2* (95% CI: 1.15, 1.46, *p* < 0.001). In *Model 3* and *Model 4*, the association was further attenuated to 1.21 and 1.19 higher odds, respectively, but remained significant (*Model 3*: 95% CI: 1.06, 1.39, *p* = 0.007; *Model 4*: 95% CI, 1.04, 1.37, *p* = 0.014). These results support hypothesis 1, indicating that a higher number of health-risk lifestyle behaviours was significantly associated with increased odds of developing dementia up to 10 years later. The LBI-sex interaction term included in the main logistic regression model was non-significant (p = 0.171), and therefore, sex-stratified analyses were not performed.

**Table 2:** Association between baseline lifestyle behaviours (wave 4) and dementia status (wave 7–9) across the 10-year study period (*N* = 3131).

Variables	Dementia status							
	Model 1^a^		Model 2^b^		Model 3^c^		Model 4^d^	
	OR (95% CI)	*P*-value	OR (95% CI)	*P*-value	OR (95% CI)	*P*-value	OR (95% CI)	*P*-value
ILB	1.35 (1.20, 1.50)	<0.001	1.29 (1.15, 1.46)	<0.001	1.21 (1.06, 1.39)	0.005	1.19 (1.04, 1.37)	0.014
Age	N/A	N/A	1.11 (1.08, 1.13)	<0.001	1.11 (1.08, 1.13)	0.001	1.11 (1.08, 1.13)	<0.001
Sex *(ref: male)*	N/A	N/A	0.91 (0.62, 1.33)	0.616	0.09 (0.60, 1.31)	0.554	0.90 (0.61, 1.33)	0.586
Ethnicity *(ref: White)*	N/A	N/A	1.25 (0.29, 5.39)	0.762	1.28 (0.30, 5.52)	0.740	1.30 (0.30, 5.63)	0.725
Marital status, *(ref: married/remarried*	N/A	N/A						
Divorced/legally separated	N/A	N/A	1.13 (0.61, 2.09)	0.707	0.95 (0.50, 1.80)	0.865	0.93 (0.49, 1.76)	0.824
Widowed	N/A	N/A	1.63 (0.99, 2.67)	0.055	1.54 (0.93, 2.55)	0.097	1.48 (0.89, 2.47)	0.131
Single	N/A	N/A	1.02 (0.45, 2.30)	0.954	0.94 (0.41, 2.11)	0.871	0.97 (0.43, 2.20)	0.949
Total household wealth, *(ref*: *1*^*st*^*quintile*, *lowest)*	N/A	N/A	N/A	N/A				
2^nd^ quintile	N/A	N/A	N/A	N/A	0.67 (0.37, 1.23)	0.193	0.67 (0.37, 1.23)	0.198
3^rd^ quintile	N/A	N/A	N/A	N/A	0.58 (0.31, 1.08)	0.084	0.60 (0.32, 1.12)	0.109
4^th^ quintile	N/A	N/A	N/A	N/A	0.67 (0.35, 1.26)	0.212	0.68 (0.35, 1.29)	0.235
5^th^ quintile (highest)	N/A	N/A	N/A	N/A	0.41 (0.21, 0.82)	0.011	0.41 (0.21, 0.82)	0.012
Education level, *(ref: low)*	N/A	N/A	N/A	N/A				
Intermediate	N/A	N/A	N/A	N/A	1.36 (0.88, 2.30)	0.172	1.36 (0.88, 2.11	0.170
Higher	N/A	N/A	N/A	N/A	0.91 (0.52–1.59)	0.729	0.90 (0.51, 1.59)	0.721
BMI (kg/m^2^) *(ref: UW/NW,* < *24.9)*	N/A	N/A	N/A	N/A	N/A	N/A		
Overweight (25.0–29.9)	N/A	N/A	N/A	N/A	N/A	N/A	1.20 (0.77, 1.88)	0.423
Obese (≥30.0)	N/A	N/A	N/A	N/A	N/A	N/A	0.82 (0.49, 1.39)	0.463
CES-D Depressive Symptoms *(ref: normal, CES-D score* < *3)*	N/A	N/A	N/A	N/A	N/A	N/A	1.44 (0.91, 2.26)	0.117
CHD *(ref: no)*	N/A	N/A	N/A	N/A	N/A	N/A	0.91 (0.45, 1.84)	0.797
Hypertension *(ref: no)*	N/A	N/A	N/A	N/A	N/A	N/A	1.21 (0.80, 1.85)	0.369
Stroke *(ref: no)*	N/A	N/A	N/A	N/A	N/A	N/A	0.94 (0.26, 3.39)	0.920

*Note.* CI= Confidence intervals; OR= Odds ratio; ILB = Index of lifestyle behaviours; BMI= Body mass index; CES-D = Centre for Epidemiologic Studies Depression Scale; UW= Underweight; NW= Normal weight; CHD= Coronary heart disease; N/A = Not applicable.

a Model 1: unadjusted effect estimates.

b Model 2: Model 1 & further adjusted for demographic factors (age, sex, ethnicity, marital status).

c Model 3: Model 2 & further adjusted for socioeconomic variables (total household wealth, education).

d Model 4: Model 3 & further adjusted for BMI and health conditions (CHD, hypertension, stroke, depression).

### 4.3 Association between lifestyle behaviours and dementia status via C-reactive protein

**Fig. 3:**
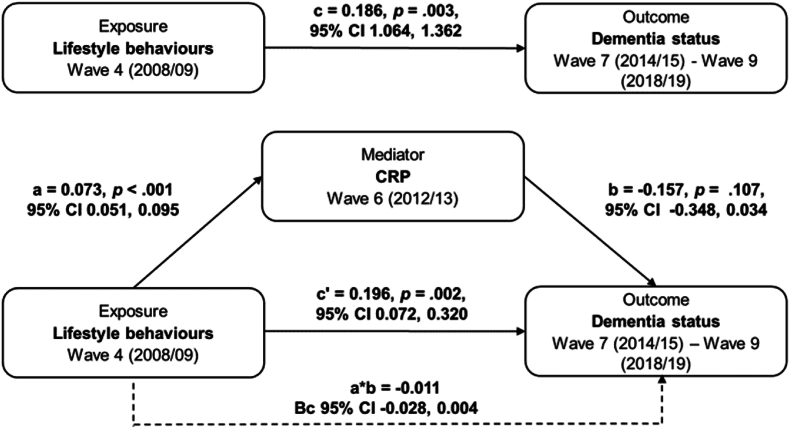
Fully adjusted mediation model of the association between lifestyle behaviours (wave 4) and dementia status (wave 7–9) via C-reactive protein (wave 6) (*N* = 3131) *Note*. CRP= C-reactive protein; CI= Confidence intervals; Bc = Bias-corrected bootstrap Mediation analyses were adjusted for sociodemographic (age, sex, ethnicity, marital status), socioeconomic (total household wealth, education) and health-related (coronary heart disease, hypertension, stroke, depression) variables, and body mass index. Due to the dichotomous outcome (dementia status), the unstandardised coefficients *b, c, c'* and a*b are logistic regression coefficients displayed on a log-odds metric. Only the unstandardised coefficient *a* represents a linear regression coefficient estimating the association between the ILB and CRP as continuous outcome. The indirect effect was quantified using bias-corrected 95% confidence intervals based on 1000 bootstrap samples.

Fig. 3 illustrates the results of the maximally adjusted mediation analysis. Contrary to hypothesis 2, CRP did not mediate the association between the ILB and dementia incidence (indirect effect a*b: B = −0.011, Bc 95% CI: −0.028 0.004). Higher scores on the ILB were significantly associated with higher CRP levels up to five years later (path *a*: B = 0.073, 95% CI: 0.051, 0.095, *p* < 0.001). However, CRP levels were not associated with higher rates of incident dementia across the subsequent seven years (path *b*: B = −0.157, 95% CI:

−0.348, 0.034, *p* = 0.107). The total effect of the ILB on dementia was positive and significant (total effect *c*: B = 0.186, 95% CI: 1.064, 1.362, *p* = 0.003) and did not change significantly after controlling for CRP at wave 6 (direct effect c': B = 0.196, 95% CI: 0.072, 0.320, *p* = 0.002). The index of the moderated mediation (index: −0.001, Bc 95% CI: −0.006 0.005) was non-significant and therefore, sex-stratified analyses were not performed.

### 4.4 Sensitivity analyses results

In the first sensitivity analysis, adjusting the original mediation analysis for baseline CRP levels produced no significant changes in the original results (see Supplementary Material Fig. S1). The indirect effect remained non-significant, implying no mediating effect of CRP (Bc 95% CI: −0.009, 0.013). The ILB was significantly positively related to subsequent CRP levels (*p* < 0.001), but there was no evidence of an association between CRP levels and subsequent dementia status (*p* = 0.721). The total effect was significantly positive (*p* = 0.007), as was the direct effect (*p* = 0.007).

Similarly, the results of the second sensitivity analysis, which included participants with CRP values ≥ 10 mg/L, mirrored the results of the original mediation model (see Supplementary Material Fig. S2). The indirect effect remained non-significant (Bc 95% CI: −0.013, 0.009). While there was a positive association between the ILB and CRP levels (*p* < 0.001), CRP levels were not associated with dementia (*p* = 0.924). Both the total effect (*p* = 0.022) and the direct effect were significantly positive (*p* = 0.021).

The third sensitivity analysis, stratified by age, showed no significant differences in results for middle-aged participants (*N* = 2274) compared to the original mediation results (see Supplementary Material Fig. S3). Results showed a positive association between the ILB and CRP levels (*p* < 0.001) but no association between CRP levels and dementia (*p* = 0.918). The indirect effect was non-significant (Bc 95% CI: −0.021, 0.023), while the total effect (*p* = 0.008) and the direct effect were both significantly positive (*p* = 0.011). By contrast, for older participants (*N* = 857), all paths and effects were non-significant (see Supplementary Material Fig. S4). The ILB was not significantly associated with CRP (*p* = 0.727), and neither was CRP with dementia (*p* = 0.130). The indirect (Bc 95% CI: −0.009, 0.015), total (*p* = 0.174), and direct effect (*p* = 0.130) were all non-significant.

In the final sensitivity analysis, adjusting the original mediation analysis for diabetes yielded no significant alterations in the original results (see Supplementary Material Fig. S5). The indirect effect continued to be non-significant, indicating the absence of a mediating effect of CRP (Bc 95% CI: −0.032, 0.008). The ILB demonstrated a positive association with subsequent CRP levels (*p* < 0.001); however, no association was observed between CRP levels and subsequent dementia status (*p* = 0.215). The total effect was significantly positive (*p* = 0.015), as was the direct effect (*p* = 0.011).

## 5 Discussion

This longitudinal study established an association between concurrent engagement in several lifestyle behaviours and subsequent dementia incidence in a large, nationally representative sample of older English adults. As hypothesised, a higher number of health-risk behaviours was significantly associated with increased odds of developing dementia up to 10 years later, independent of important covariates such as wealth, BMI, CHD, hypertension, stroke, and depression. Sensitivity analyses 1 and 4 demonstrated that the significant association between health-risk behaviours and subsequent dementia status persisted after adjusting for baseline CRP levels and dementia, respectively.

The behaviour-dementia association was not mediated by inflammation as we originally hypothesised. While lifestyle behaviours were linked to subsequent CRP levels, increased CRP levels were not linked to a higher incidence of dementia and did not mediate the association between lifestyle and subsequent dementia risk. Sensitivity analysis 2 showed that the non-significant mediation effect was not due to the exclusion of participants with CRP values exceeding 10 mg/L.

To the best of our knowledge, this study was the first to establish the combined impact of lifestyle behaviours on dementia incidence in a UK cohort. Thereby, it extends previous US-based findings on the associations between co-occurring lifestyle behaviours and subsequent AD (Dhana et al., 2020; Norton et al., 2012; Scarmeas et al., 2009) and all-cause dementia risk (Norton et al., 2012) to the UK and other peer countries. Although the effect sizes were relatively small (Lu and Chen, 2018), given the high lifestyle risk burden in the population, such modest individual-level effects could substantially reduce dementia incidence at the population-level (Wolters et al., 2019). In practice, the ILB could be used to identify at-risk populations and to distribute easily comprehensible information about behavioural risk.

Notably, the present age-stratified sensitivity analyses showed that the ILB was only predictive of dementia status in middle-aged but not older adults. Similarly, a previous lifestyle index comprising behavioural and cardiometabolic factors was associated with an increased risk of dementia when it was measured in middle-aged (40–50 years) but not in older participants (65–79 years) (Deckers et al., 2020). These age-dependent results might be due to older participants being classified as dementia cases during follow-up, having undiagnosed and presumably irreversible neuropathology at baseline (Narayanan and Murray, 2016). Such neuropathology would not be amendable through lifestyle modification, indicating that lifestyle modification might only be effective in preventing dementia if started in mid-life. Nevertheless, further longitudinal validation of the ILB in age-stratified samples is warranted as the present null findings might have been caused by differential (non-random) enrolment and attrition of older participants (Wang and Kattan, 2020). Older participants with a high-risk burden commonly refrain or drop out from participation due to ill health, disability, or death before they could have developed dementia (Jacobsen et al., 2021). Such differential participant enrolment and attrition could attenuate or even reverse true associations between harmful exposures and disease outcomes (Weuve et al., 2015), potentially explaining the present null findings in older participants.

Despite promising empirical background and biological plausibility, the current results did not support the hypothesised mediating role of inflammation in the relationship between health-risk behaviours and dementia. It is plausible that this association might be determined by alternative or additional biological effects of behaviours on brain health (e.g., cardiovascular damage, neurodegeneration, hypoxia) (Grande et al., 2020; Livingston et al., 2020).

In the current study, lifestyle behaviours predicted CRP levels, suggesting that the non-significant mediation through CRP is likely to be attributable to the absence of a predictive role of CRP in dementia. The lack of CRP-dementia association conflicts with several studies that linked higher levels of inflammatory markers, including CRP, with increased risk of all-cause dementia up to 25 years later (e.g., Hsu et al., 2017). The most recent meta-analysis confirmed these prospective links, demonstrating that the risk of all-cause dementia was increased by elevated CRP levels across eight longitudinal studies (Darweesh et al., 2018). However, consistent with the present absence of a CRP-dementia association, several studies have not demonstrated an association between CRP levels and subsequent all-cause dementia incidence (e.g., Himbergen et al., 2012).

One interpretation for these null findings pertains to the potential differential predictive value of CRP for different dementia subtypes. Two meta-analyses found associations between CRP and VAD, but evidence for an association with AD was minimal (Koyama et al., 2013) or non-existent (Darweesh et al., 2018). As dementia subtypes are combined for all-cause dementia outcomes, current and previous null findings for all cause-dementia could be due to potential non-significant CRP-AD associations masking existing associations between CRP and other dementia subtypes (e.g., VAD). Such masking effects are plausible since individuals with pure AD make up a large proportion (30–40%) of dementia cases (DeTure and Dickson, 2019). Additional research should investigate the effects of CRP on different dementia subtypes.

A further interpretation of the current absence of a CRP-dementia association concerns the potential U-shaped pattern of CRP levels across dementia development. Research suggests that CRP levels might be heightened decades before dementia onset and during its severe phase, while they might be lower during the immediate years before clinical symptom manifestation (Fernandes et al., 2020). In the present study, dementia ascertainment started one year after the CRP measurement. Thus, during the CRP measurement, participants developing dementia might have been in the prodromal stage of the syndrome, which has been associated with lowered CRP levels, potentially explaining the current null findings (Fernandes et al., 2020; Gong et al., 2016). In support of this, prior studies with shorter follow-ups found no prospective CRP-dementia associations (e.g., Eriksson et al., 2011; Kravitz et al., 2009), while those with longer follow-ups (13–25 years) showed such associations (e.g., Schmidt et al., 2002; Tao et al., 2018). Given the potential decade-long neuropathological development of dementia (Vermunt et al., 2019), multiple CRP measurements over prolonged time periods (>20 years) will be required in future research to clarify the potential U-shaped pattern of CRP trajectories over the course of dementia.

A noteworthy finding is the prospective link between lifestyle behaviours and CRP levels, extending the previously established cross-sectional associations (Bonaccio et al., 2017; Loprinzi, 2016). The influence of lifestyle behaviours on inflammation is biologically plausible. For instance, disrupted sleep (Irwin et al., 2016), cigarette smoking (Strzelak et al., 2018), and social isolation (Smith et al., 2020) can upregulate the production of pro-inflammatory cytokines while physical activity and a wholefood, plant-rich diet, and low-to-moderate alcohol consumption can exert anti-inflammatory effects by improving insulin sensitivity, endothelial function, and cholesterol metabolism (Pedersen, 2017; Tuttolomondo et al., 2019). In line with this behavioural evidence, our findings suggest that mid-life modification of health-risk behaviours might slow or attenuate inflammageing, a desirable outcome linked to reduced risk of chronic disease, frailty, and premature death (Ferrucci and Fabbri, 2018). The potential anti-inflammatory effects of multi-behaviour interventions should be tested in future RCTs.

### 5.1 Limitations

The current findings should be interpreted in light of various limitations. Although this observational study benefitted from extensive covariate adjustment, the risk of residual confounding cannot be ruled out, and causality cannot be inferred. Reverse causality bias was minimised through a 10-year follow-up and the exclusion of participants who developed dementia three to five years after baseline. Nevertheless, given the long subclinical phase of dementia, it remains possible that lifestyle behaviours and CRP levels were affected by subclinical dementia processes (Sattar and Preiss, 2017). While measurement validity was maximised through expert consensus, prior research, and extensive piloting (Marmot et al., 2003), the measurement of lifestyle behaviours was based on rather crude and subjective reporting, introducing measurement imprecision and potential recall or social desirability bias. Additional research would benefit from a detailed and comprehensive evaluation of behavioural variables, such as employing a food frequency questionnaire to assess diet alongside objective assessments such as pedometers or accelerometers to assess physical activity levels. A further limitation is the measurement of inflammation - a system of interacting, heavily intertwined components (Furman et al., 2019), which we might not have captured by limiting assessment to CRP. CRP is a protein that has recently been identified as a marker of somatic maintenance rather than inflammation specifically (Del Giudice and Gangestad, 2018). For a more accurate assessment of inflammation, future studies should include several unambiguous inflammatory biomarkers such as IL-1β and TNF-α (Bennett et al., 2018), but these were not available in ELSA. Another drawback is the use of unweighted behavioural variables, which may neglect the relative importance of each behaviour within the overall behavioural profile (Livingston et al., 2020). Additional precision by weighting each behaviour would be desirable in future research. Lastly, the present study participants appeared more affluent, educated, and slightly healthier than the target population (i.e., older English adults). Nonetheless, the presently estimated exposure-disease associations are likely generalisable to the target population as their accuracy is not reliant upon population-level representativeness (Fry et al., 2017).

## 6 Conclusion

In a large prospective cohort of English community-dwelling older adults, we found that engaging in a higher number of health-risk behaviours was associated with increased incident dementia up to 10 years later, but it was not explained by inflammation, implying that other biological pathways might be at play. These findings suggest that the cumulation of daily lifestyle choices represents a robust predictor of dementia and should be targeted for its modifiable nature in dementia prevention.

## Funding

The English Longitudinal Study of Ageing is funded by the 10.13039/100000049National Institute on Aging (Grant RO1AG7644) and a consortium of UK government departments coordinated by the 10.13039/501100000269Economic and Social Research Council (10.13039/501100000269ESRC) and the Office for National Statistics. The English Longitudinal Study of Ageing (ELSA) is managed by a team of researchers based at University College London, the Institute for Fiscal Studies, and the National Centre for Social Research. The data are linked with the UK Data Archive and are freely available through the UK data services can be accessed here: https://discover.ukdataservice.ac.uk (reference number 10.5255/UKDA-SN-8444-2). DC is funded by the 10.13039/501100000143Alzheimer Society (Grant 407), 10.13039/501100000268BBSRC, Department of Health, 10.13039/501100000269Economic and Social Research Council (10.13039/501100000269ESRC) (grants ES/T012091/1 & ES/S013830/1), NIHR and UKHSA. PF is funded by 10.13039/100010269Wellcome Trust (221854/Z/20/Z).

## CRediT authorship contribution statement

**Leah Hillari:** Conceptualization, Formal analysis, Investigation, Methodology, Project administration, Visualization, Writing – original draft, Writing – review & editing. **Philipp Frank:** Methodology, Project administration, Supervision, Writing – review & editing. **Dorina Cadar:** Conceptualization, Data curation, Formal analysis, Funding acquisition, Investigation, Project administration, Supervision, Validation, Writing – review & editing.

## Declaration of competing interest

There is no conflict of interest among all authors.

## Data Availability

The authors do not have permission to share data.
