# Supplementary material for: Systemic inflammation, lifestyle behaviours and dementia: A 10-year follow-up investigation
Source: Brain Behav Immun Health. 2024 Apr 22;38:100776. doi: 10.1016/j.bbih.2024.100776 (PMC11068506; doi:10.1016/j.bbih.2024.100776)
Supplement: Multimedia component 1 [file mmc1.docx]

**Supplementary Information**

**Table S1.** Classification of lifestyle behaviours for the creation of the index of lifestyle behaviours

| **Lifestyle behaviour** | **Health-protective level (0)** | **Health-risk level (1)** | **The rationale for the classification of behaviours** |
| --- | --- | --- | --- |
| Alcohol intake | Low-to-moderate alcohol intake:  1-14 alcoholic drinks per week | No or excessive alcohol intake:  no alcohol intake  or >14 alcoholic drinks per week | Meta-analytic and systematic review evidence demonstrated the protective effect of low-to-moderate alcohol intake on brain health (Anstey et al., 2009; Peters et al., 2008; Xu et al., 2017), typically defined as 1–14 standard drinks per week in line with the current UK alcohol guidelines (Department of Health, 2016). |
| Fruit & vegetable consumption | Adequate fruit & vegetable consumption: ≥5 portions of fruit & vegetables per day | Insufficient fruit & vegetable consumption: <5 portions of fruit & vegetables per day | Consuming at least 5 portions (≥400 grams) of fruit and vegetables daily is recommended by the National Health Service (NHS, 2018) and international guidelines (WHO, 2020) to maintain health and reduce disease risk (Wang et al., 2021) |
| Smoking | Not currently smoking (never/past smoking) | Currently smoking | Meta-analytic evidence found current smokers at increased risk of dementia compared to never and former smokers (Zhong et al., 2015; Peters et al., 2008; Anstey et al., 2007). |
| Physical activity | Physically active:  ≥1/week moderate or vigorous physical activity | Physically inactive:  1-3 times a month moderate or vigorous physical activity or hardly ever/never moderate or vigorous physical activity | The classification was based on recommendations for older adults to engage in physical activity at least 1 hour per week to lower dementia risk (Lee, 2018) and broadly mapped onto the physical activity recommendations by the UK Chief Medical Officers of 75 minutes of vigorous-intensity or 150 minutes of moderate-intensity activity per week (Davies et al., 2019). |
| Sleep duration | Adequate sleep duration: ≥7 hours average sleep duration on a weeknight | Short sleep duration:  <7 hours average sleep duration on a weeknight | Sleeping at least 7 hours per night is recommended by the Sleep Research Society and the American Academy of Sleep Medicine (Watson et al., 2019) and the National Sleep Foundation (Hirshkowitz et al., 2015) to promote optimal health and function. |
| Social engagement^†^ | Socially engaged: Engagement in ≥3 of 7 social leisure activities (highest 40% of the distribution) | Socially disengaged: Engagement in <3 of 7 social leisure activities (lowest 60% of the distribution) | Cut-off based on the 60th percentile |
| Cognitive activity^‡^ | Cognitively active: Engagement in ≥3 of 6 intellectual leisure activities (highest 40% of the distribution) | Cognitively inactive: Engagement in <3 of 6 intellectual leisure activities (lowest 60% of the distribution) | Cut-off based on the 60th percentile |

*Note.* ^†^ The social engagement score consisted of seven social leisure activities: looking after others; belonging to a group (e.g., environmental, political, neighbourhood); being a member of a gym, exercise class or sports club; being part of a church/religious group; belonging to a social club and/or meeting with friends; engaging with a charitable association; and having taken holidays and/or day trips within the last 12 months. ^‡^The cognitive activity score was composed of six intellectual leisure activities: attending evening classes or arts, music, or education groups; owning a mobile phone; using the internet or email; reading the newspaper daily; having a hobby or pastime; and cultural engagement (visiting a gallery/museum, theatre, concert/opera, or a cinema).

**Detailed Explanation of Sensitivity Analyses**

Several sensitivity analyses were conducted to test the robustness of the findings. The first sensitivity analysis re-estimated the initial mediation model, including baseline CRP levels as a covariate. The main analysis did not adjust for baseline CRP levels, as lifestyle behaviours are anticipated to exert an immediate impact on inflammatory levels at the time of measurement rather than five years later. Consequently, controlling for baseline inflammation might cancel out some of the initial effects of lifestyle behaviours on inflammatory levels and bias the results towards the null. Nevertheless, including a sensitivity analysis which controls for the mediator at baseline is recommended to ensure the temporal precedence of the exposure (lifestyle behaviours) relative to the mediator (inflammation), allowing causal inference about the assumed effect of the exposure on the mediator. To test this temporal precedence of the exposure, the initial mediation model was re-estimated adjusting for baseline CRP levels.

In the second sensitivity analysis, the main mediation model was re-estimated, including participants with CRP values ≥10 mg/L. While CRP values ≥10 mg/L have been conventionally treated as an indication of acute infection/injury, accumulating evidence suggests that such elevated CRP levels can be observed even with chronic systemic inflammation (Mac Giollabhui et al., 2020).

In the third sensitivity analysis, the initial mediation model was stratified by age, estimating the model separately for participants who were in their mid-life (50-65 years) and for those who were in their later-life (>65 years) during exposure measurement. Lifestyle factors have shown age-dependent effects (Deckers et al., 2020). Thus, age stratification was deemed important to compare the relative predictive validity of the ILB and the potential mediating role of inflammation in middle-aged and older individuals.

In the last sensitivity analysis, the initial mediation model was replicated, including baseline diabetes status as a covariate. Diabetes is a critical risk factor for dementia (Livingston et al., 2020) and has been consistently associated with lifestyle behaviour (Khan et al., 2023) and inflammation (Wang et al., 2013).

*
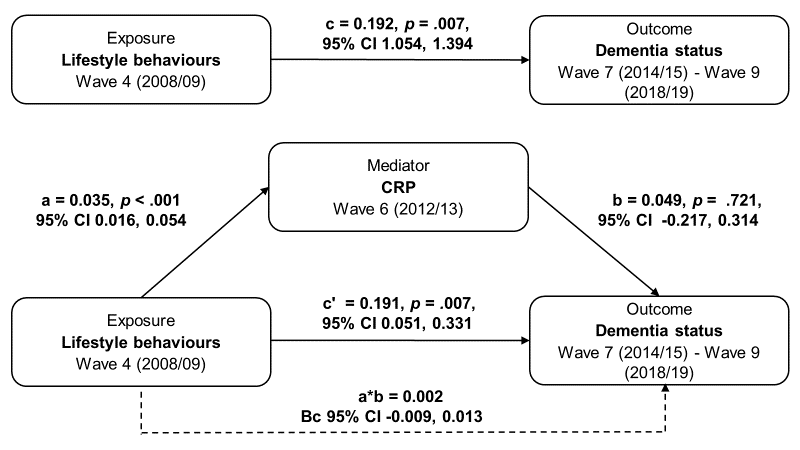
*

**Figure S1** Fully adjusted mediation model of the association between lifestyle behaviours (wave 4) and dementia status (wave 7-9) via c-reactive protein (wave 6) adjusting for baseline c-reactive protein (wave 4) (N= 3,313)

*Note*. CRP= C-reactive protein; CI= Confidence intervals; Bc= Bias-corrected bootstrap

Mediation analyses were adjusted for sociodemographic (age, sex, ethnicity, marital status), socioeconomic (total household wealth, education) and health-related (coronary heart disease, hypertension, stroke, depression) variables, body mass index, and baseline CRP levels.

Due to the dichotomous outcome (dementia status), the unstandardised coefficients *b, c, c’* and *a*b* are logistic regression coefficients displayed on a log-odds metric. Only the unstandardised coefficient *a* represents a linear regression coefficient estimating the association between the ILB and CRP as a continuous outcome. The indirect effect was quantified using bias-corrected 95% confidence intervals based on 1,000 bootstrap samples.

**
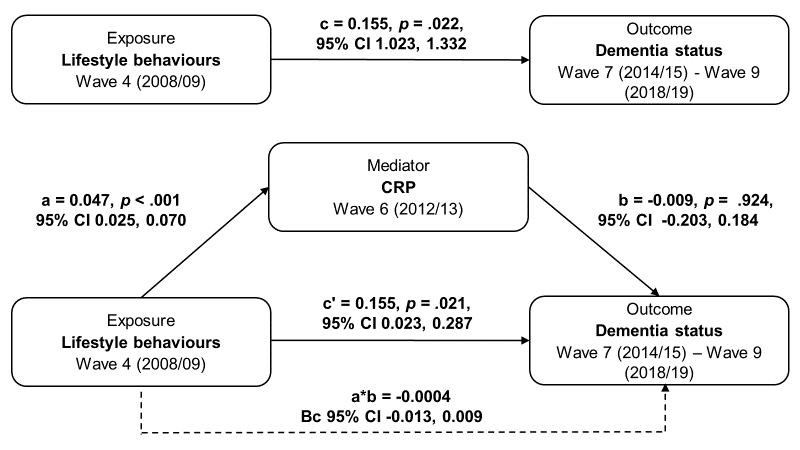
**

**Figure S2** Fully adjusted mediation model of the association between lifestyle behaviours (wave 4) and dementia status (wave 7-9) via c-reactive protein (wave 6) including cases with c-reactive protein ≥10 mg/L (wave 4 and 6) (N= 3,444)

*Note*. CRP= C-reactive protein; CI= Confidence intervals; Bc= Bias-corrected bootstrap

Mediation analyses were adjusted for sociodemographic (age, sex, ethnicity, marital status), socioeconomic (total household wealth, education) and health-related (coronary heart disease, hypertension, stroke, depression) variables, and body mass index.

Due to the dichotomous outcome (dementia status), the unstandardised coefficients *b, c, c’* and *a*b* are logistic regression coefficients displayed on a log-odds metric. Only the unstandardised coefficient *a* represents a linear regression coefficient estimating the association between the ILB and CRP as a continuous outcome. The indirect effect was quantified using bias-corrected 95% confidence intervals based on 1,000 bootstrap samples.


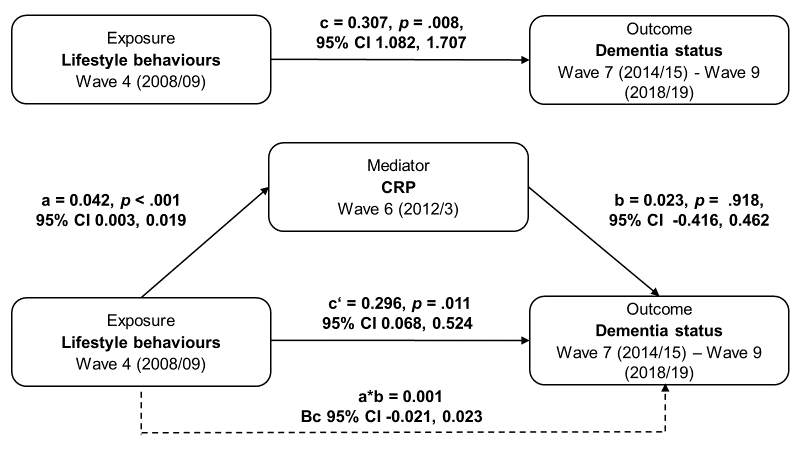


**Figure S3** Fully adjusted mediation model of the association between lifestyle behaviours (wave 4) and dementia status (wave 7-9) via c-reactive protein (wave 6) in participants aged 50-65 years (N= 2,274)

*Note*. CRP = C-reactive protein; CI = Confidence interval; Bc = Bias-corrected bootstrap

Mediation analyses were adjusted for sociodemographic (age, sex, ethnicity, marital status), socioeconomic (total household wealth, education) and health-related (coronary heart disease, hypertension, stroke, depression) variables, and body mass index.

Due to the dichotomous outcome (dementia status), the unstandardised coefficients *b, c, c’* and *a*b* are logistic regression coefficients displayed on a log-odds metric. Only the unstandardised coefficient *a* represents a linear regression coefficient estimating the association between the ILB and CRP as a continuous outcome. The indirect effect was quantified using bias-corrected 95% confidence intervals based on 1,000 bootstrap samples.

*
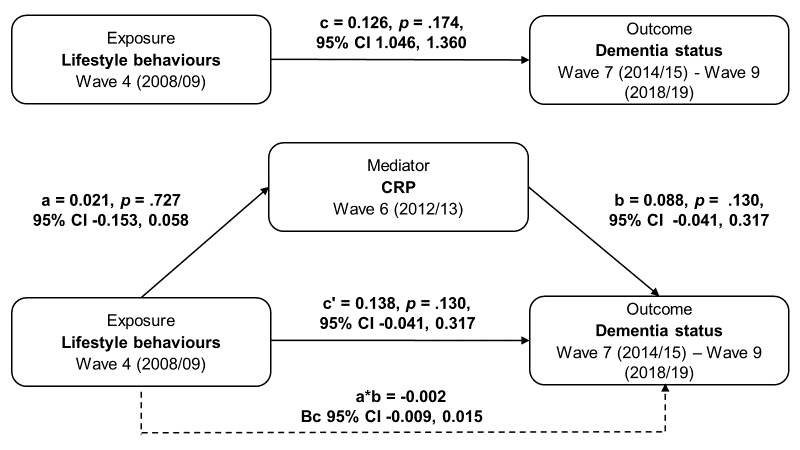
*

**Figure S4** Fully adjusted mediation model of the association between lifestyle behaviours (wave 4) and dementia status (wave 7-9) via c-reactive protein (wave 6) in participants aged >65 years (N= 857)

*Note*. CRP = C-reactive protein; CI = Confidence interval; Bc = Bias-corrected bootstrap

Mediation analyses were adjusted for sociodemographic (age, sex, ethnicity, marital status), socioeconomic (total household wealth, education) and health-related (coronary heart disease, hypertension, stroke, depression) variables, and body mass index.

Due to the dichotomous outcome (dementia status), the unstandardised coefficients *b, c, c'* and *a*b* are logistic regression coefficients displayed on a log-odds metric. Only the unstandardised coefficient *a* represents a linear regression coefficient estimating the association between the ILB and CRP as a continuous outcome. The indirect effect was quantified using bias-corrected 95% confidence intervals based on 1,000 bootstrap samples.


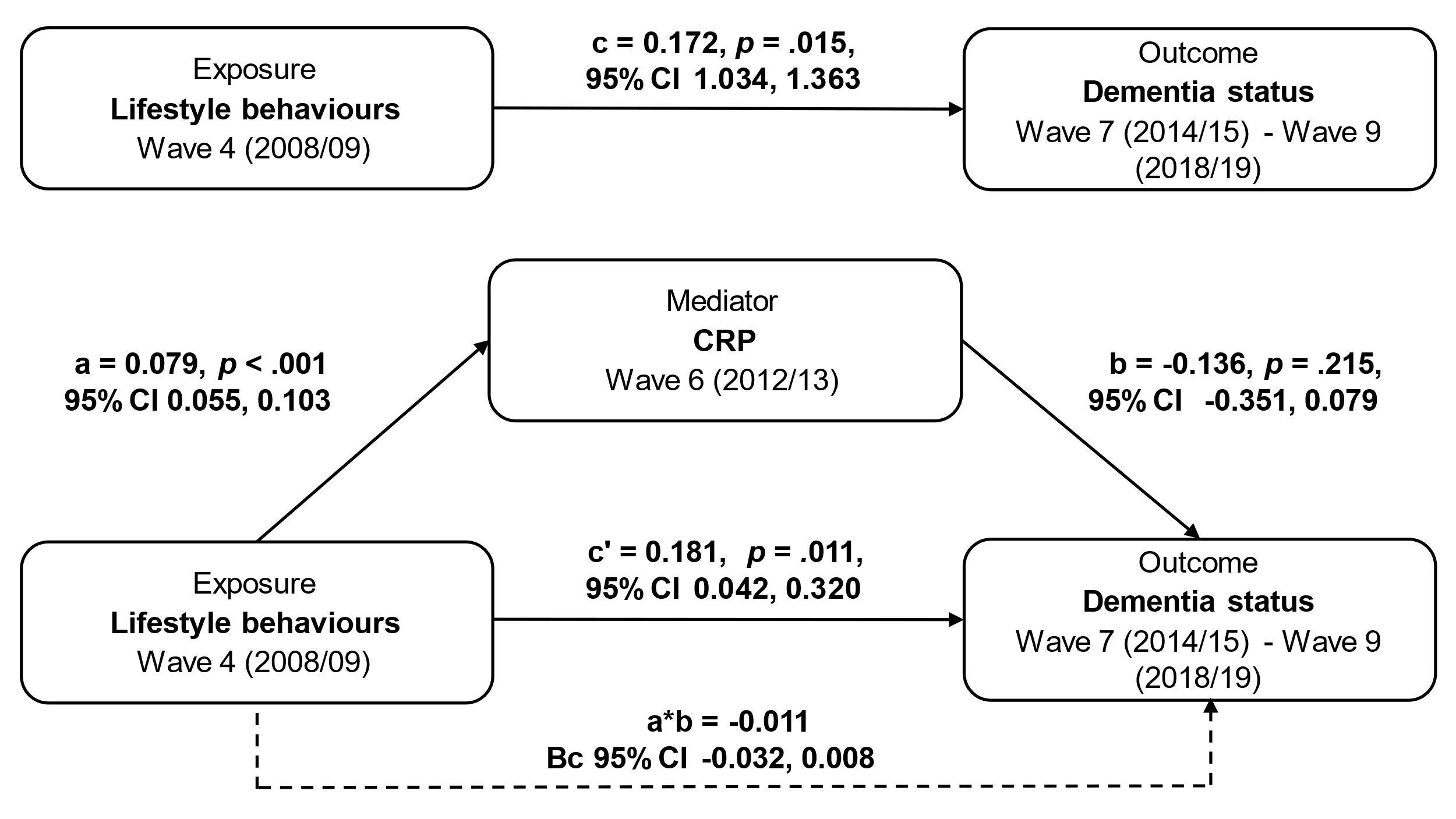


**Figure S5** Fully adjusted mediation model of the association between lifestyle behaviours (wave 4) and dementia status (wave 7-9) via c-reactive protein (wave 6) adjusting for baseline diabetes status (wave 4) (N= 3,313)

*Note*. CRP= C-reactive protein; CI= Confidence intervals; Bc= Bias-corrected bootstrap

Mediation analyses were adjusted for sociodemographic (age, sex, ethnicity, marital status), socioeconomic (total household wealth, education) and health-related (coronary heart disease, hypertension, stroke, depression) variables, body mass index, and baseline dementia status.

Due to the dichotomous outcome (dementia status), the unstandardised coefficients *b, c, c’* and *a*b* are logistic regression coefficients displayed on a log-odds metric. Only the unstandardised coefficient *a* represents a linear regression coefficient estimating the association between the ILB and CRP as a continuous outcome. The indirect effect was quantified using bias-corrected 95% confidence intervals based on 1,000 bootstrap samples.
